# Regulation of mitotic chromosome architecture and resolution of ultrafine anaphase bridges by PICH

**DOI:** 10.1080/15384101.2021.1970877

**Published:** 2021-09-16

**Authors:** Primrose Chanboonyasitt, Ying Wai Chan

**Affiliations:** School of Biological Sciences, The University of Hong Kong, Pokfulam, Hong Kong

**Keywords:** PICH, PLK1, chromosome segregation, ultrafine anaphase bridges, BLM, topoisomerase IIIα

## Abstract

To ensure genome stability, chromosomes need to undergo proper condensation into two linked sister chromatids from prophase to prometaphase, followed by equal segregation at anaphase. Emerging evidence has shown that persistent DNA entanglements connecting the sister chromatids lead to the formation of ultrafine anaphase bridges (UFBs). If UFBs are not resolved soon after anaphase, they can induce chromosome missegregation. PICH (PLK1-interacting checkpoint helicase) is a DNA translocase that localizes on chromosome arms, centromeres and UFBs. It plays multiple essential roles in mitotic chromosome organization and segregation. PICH also recruits other associated proteins to UFBs, and together they mediate UFB resolution. Here, the proposed mechanism behind PICH’s functions in chromosome organization and UFB resolution will be discussed. We summarize the regulation of PICH action at chromosome arms and centromeres, how PICH recognizes UFBs and recruits other UFB-associated factors, and finally how PICH promotes UFB resolution together with other DNA processing enzymes.

## Introduction

Equal distribution of the replicated genome into two daughter cells is crucial in maintaining genome stability and cell proliferation. It requires proper condensation and compaction of chromosomes, stepwise removal of chromosome cohesion and resolution of any persistent sister intertwining structures generated during DNA replication and repair [1–3]. Both chromosome cohesion and sister intertwinements physically connect the two sister chromatids, and therefore lead to chromosome missegregation if they are not timely removed before anaphase. Chromosome missegregation is proposed to induce chromosomal instability (CIN), a hallmark of solid tumors [4,5]. Two well-studied forms of chromosome segregation defects are lagging chromosomes and chromatin bridges. Lagging chromosomes can be induced by merotelic attachments in which a single kinetochore is attached to microtubules from the two spindle poles [6]. Chromatin bridges can be induced by chromosome fusions [7,8], defects in chromosome condensation [3,9] and failure to completely remove cohesin complexes from chromosomes [10–12]. In around a dozen of years ago, a breakthrough in the chromosome segregation field came from the discovery of a novel form of DNA bridges, which is known as ultrafine anaphase bridges (UFBs) [13,14]. UFBs have escaped detection over a long period of time as they are fine DNA threads that cannot be visualized using conventional DNA dyes and they are devoid of histones. Importantly, many CIN cancer cells have been shown to display an elevated frequency of UFBs [15]. Furthermore, Bloom syndrome (BS) patients are predisposed to all types of cancer [16] and cells derived from BS patients, that are defective in BLM protein, exhibit an elevated frequency of UFBs [14]. These observations suggest that UFBs can be a driver of CIN and tumorigenesis.

UFBs arise from persisted DNA intertwining structures between sister chromatids. Such DNA interlinks include double-stranded DNA (dsDNA) catenation, incomplete replicated DNA and Holliday junction (HJ)-like recombination intermediates [1,2,17]. Interestingly, inhibition of condensin complex has also been shown to increase the amount of UFBs [9], suggesting that proper chromosome compaction is involved in either counteracting the formation of UFBs or facilitating the removal of topological linkages between sister chromatids.

PICH (PLK1-interacting checkpoint helicase), also known as ERCC6L, is a SNF2 family member of ATPase that possesses dsDNA translocase activity [13,18,19]. SNF2 proteins belong to a family of ATPase/helicase-like proteins harboring a SNF2 domain that can convert energy from ATP hydrolysis into mechanical force to remodel chromatin structure [20]. Most SNF2 proteins are translocases that remodel nucleosomes or DNA-protein complexes. Since chromatin remodeling at DNA damage sites is required for DNA repair machinery to access, multiple SNF2 members were shown to be involved in recognizing DNA damage and facilitating the downstream repair [20].

**Figure 1.**
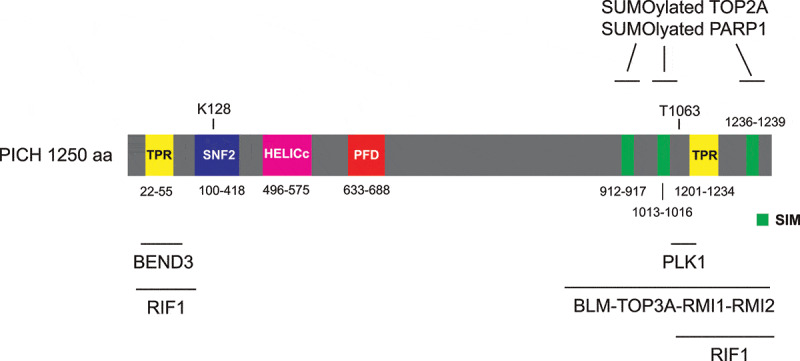
Schematic representation of the domain structure of human PICH protein

PICH harbors a SNF2-type ATPase domain that mediates ATP hydrolysis for DNA translocation, and a helicase superfamily HELICc domain in its N-terminus (Figure 1). PICH also contains two tetratricopeptide repeats (TPRs), one in the N-terminus and the other in the C-terminus, which are known to mediate protein-protein interaction. PICH harbors a conserved PICH family domain (PFD) that is specific for PICH orthologs (Figure 1). The function of PICH-PFD is currently unknown. PICH was first identified as a PLK1-interacting protein in mitosis [13,21]. PICH-PLK1 interaction in mitosis requires CDK1-mediated phosphorylation of the Polo-box domain (PBD)-binding site at Thr-1063 on PICH [13,21]. PICH T1063A mutation abolishes the interaction between PLK1 and PICH, indicating that it is the major priming phosphorylation site for binding to PLK1-PBD. PICH has been shown to play several important roles in mitotic chromosome organization and segregation. In prometaphase, PICH and PLK1 are proposed to coordinately maintain chromosome architecture by regulating DNA topoisomerase IIα (TOP2A) [22,23]. Later in anaphase, PICH serves as a “tension sensor” that recognizes UFBs and recruits other UFB-associated factors to promote UFB resolution [18]. Here, we summarize the current knowledge on PICH functions on mitotic chromosomes and DNA bridges. We emphasize on its localization and action on chromosome arms and centromeres, how it recognizes UFBs and how it cooperates with other DNA processing enzymes to promote resolution of different types of UFBs.

PICH contains two TPR domains, a SNF2-type ATPase domain, a HELICc helicase superfamily domain and a PFD domain. Three SUMO-interacting motifs (SIMs) are also identified. K128 is responsible to bind ATP and phosphorylation at T1063 is responsible to bind to PLK1. BEND3 interacts with the N-terminal TPR domain. Interaction between RIF1 and PICH is abolished when both PICH-TPR domains are deleted. C-terminus of PICH (791–1250 aa.) interacts with the BTRR complex. Finally, the PICH SIMs are responsible for the interactions with SUMOylated TOP2A and SUMOylated PARP1.

## Localization and functions of PICH at chromosome arms

In prometaphase, PLK1 and PICH co-localize primarily at centromeres, while a subpopulation of PLK1 and PICH co-localizes on chromosome arms [13,21,23,24]. Interestingly, siRNA-mediated depletion of PLK1 leads to spreading of PICH over chromatid arms [13]. Similarly, a later study using an ATP-competitive inhibitor of PLK1, ZK-thiazolidinone (TAL), also showed that inactivation of PLK1 leads to spreading of both PLK1 and PICH over chromatid arms [24,25]. These results suggest that PLK1 activity is required for the removal of PICH from the chromosome arms. On the other hand, depletion of PICH results in a reduction of PLK1 level on chromosome arms, whereas localization of PLK1 at centromeres and spindle remains unaltered [23]. The translocase activity of PICH is required for its removal from chromosome arms as the ATPase-dead PICH spreads all over the chromosome arms and PLK1 follows the relocalization from kinetochores to chromosome arms [21,24]. Importantly, when expressing ATPase-dead PICH that cannot bind to PLK1 (by introduction of T1063A mutation), relocalization of PLK1 to chromosome arms does not occur [24]. Together, these studies showed that PICH is responsible to recruit a subpopulation of PLK1 to chromosome arms.

What is the function of PICH at the chromosome arms? PICH is proposed to maintain proper prometaphase chromosome architecture as depletion of PICH by siRNA leads to disorganized chromosomes with more opened and “wavy” arms [23]. Interestingly, inhibition of TOP2A during mitosis by ICRF-193 (Meso-4,4’-(3,2-butanediyl)-bis(2,6-piperazinedione)), an inhibitor that stalls TOP2A at the last step of the strand passing reaction and therefore traps the two DNA strands within the TOP2A molecule [26,27], prevents the chromosome disorganization induced by PICH depletion [23]. This finding raises an attractive hypothesis that the key function of PICH at chromosome arms is to suppress the TOP2A decatenation activity in prometaphase. TOP2A is the most abundant component of the chromosome scaffold. Although its activity is important for chromosome compaction and sister chromatid separation [28–32], it is possible that unconstrained TOP2A activity would lead to prolonged condensation, leading to more compacted chromosomes. It also induces premature opening of sister chromatid arms. As a result, unconstrained TOP2A activity leads to highly disorganized, wavy chromosomes [23]. Recent studies have provided the details of how PICH regulates TOP2A on chromosomes. PICH contains three SUMO-interacting motifs (SIMs) at its C-terminus (Figure 1), which mediate interaction with chromosomal SUMOylated proteins [33]. It is likely that PICH translocates along the DNA with the SUMOylated proteins, removing them from chromosomes [22,34]. SUMOylated TOP2A was proposed to be the main target of PICH on chromosomes. Increased levels of both PICH and SUMOylated TOP2A on chromosomes are observed upon ICRF-193 treatment [22,35,36], indicating that an active population of TOP2A stalled on DNA is SUMOylated and interacts with PICH. Depletion of PICH or inhibiting PICH ATPase activity lead to increased abundance of SUMOylated TOP2A on chromosomes [22], suggesting that PICH translocase activity is responsible to remove SUMOylated TOP2A from chromosomes. Furthermore, interaction of PICH with SUMOylated TOP2A reduces TOP2A decatenation activity *in vitro* [22]. These results conclude that PICH controls SUMOylated TOP2A by modulating both its chromosomal association and activity. On the other hand, PLK1 has been shown to phosphorylate TOP2A at Ser-1337 and Ser-1524, enhancing its decatenation activity [37]. Therefore, chromosome arm population of PLK1-PICH may fine-tune the activity of TOP2A for proper chromosome compaction and organization.

## Localization and functions of PICH at centromeres

PICH localizes primarily to centromeres at prometaphase, metaphase and anaphase [13]. SUMOylated TOP2A, a major centromeric SUMO substrate, contributes to the centromeric localization of PICH [22]. A fusion protein that can deSUMOylate centromeric proteins were employed to test if SUMOylation controls PICH localization at centromeres. The fusion protein contains a SENP2-catalytic domain (mediates deSUMOylation) and a N-terminus of PIASy (localizes to kinetochores). When the fusion protein was expressed in mitotic cells, it deSUMOylated TOP2A and the centromeric localization of PICH no longer increased under ICRF-193 treatment [22]. Furthermore, PICH interacts with SUMOylated poly-(ADP-ribose) polymerase 1 (PARP1, an enzyme that catalyzes PARylation of many nuclear proteins [38]) at centromeres [34]. Together, these results suggest that interaction with SUMOylated centromeric proteins via the PICH SIMs is likely to be the major mechanism of its centromeric accumulation. The reason for the need of accumulating PICH at centromeres may be because centromeric DNA remains highly catenated as centromeric cohesion, which blocks TOP2A-mediated DNA decatenation, is only removed upon anaphase onset [32,39,40]. Therefore, centromeric DNA frequently forms UFBs that require PICH for their resolution in anaphase (see below). Recently, two independent groups have reported that PICH recruitment to centromeres is negatively regulated by PLK1 activity [41,42]. Inactivation of PLK1 by an ATP-competitive inhibitor BI2536 [43], or through the inhibition of PLK1^as^, an analogue-sensitive mutant that has been used to replace the endogenous PLK1, by 3-MB-PP1 (an ATP analogue that selectively inhibits PLK1^as^) [44] increase the binding of PICH and BLM (Bloom syndrome protein) to the centromeres [41,42]. BLM is a 3’ to 5’ ATP-dependent RecQ DNA helicase that is involved in the regulation of DNA replication and recombination [45]. BLM recognizes and processes a wide variety of DNA structures, including G-quadruplex, replication intermediate and Holliday junction, etc. Depletion of PICH abolishes the kinetochore localization of BLM but not *vice versa*, indicating that PICH acts upstream to recruit BLM to centromeres. Upon PLK1 inhibition, PICH and BLM cooperate to excessively unwind centromeric DNA linkages, resulting in subsequent whole chromosome arm splitting. This previously undescribed phenomenon is termed “centromere disintegration”, which can lead to metaphase collapse [41,42]. This centromere disintegration requires BLM helicase activity as replacing wild-type BLM with a helicase-dead BLM prevents such phenotype [42]. These results concluded that PLK1 prevents excessive accumulation of PICH/BLM at centromeres. Whether PICH translocase activity is required for unwinding centromeric DNA remains to be investigated. Together, these studies suggest an intricate network of signals: PLK1 and SUMOylated TOP2A/PARP1 work together to control the level of PICH at centromeres.

**Figure 2.**
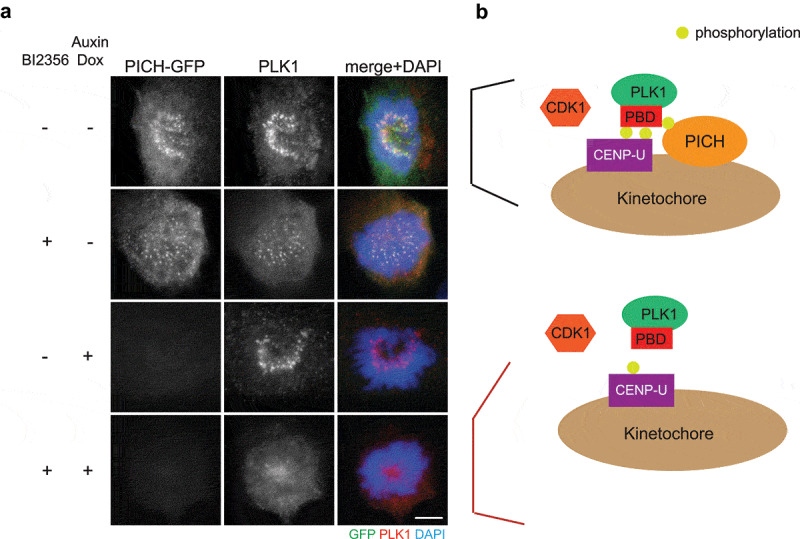
PICH depletion abolishes kinetochore PLK1 when PLK1 activity is inhibited

We propose that another function of PICH at centromeres/kinetochores is to stabilize PLK1 localization at kinetochores (Figure 2). Previous studies confirmed that PBD-binding protein 1 (PBIP1, also known as CENP-U) is the key PLK1 receptor at kinetochores [46–48]. CENP-U accumulates at interphase centromeres prior to PLK1. In prophase, PLK1 phosphorylates CENP-U at Thr-78 and CDK1 phosphorylates CENP-U at Thr-98. These two phosphorylation events create docking sites for CENP-U binding to PLK1, and therefore recruit PLK1 to kinetochores [46–48]. As previously shown, inhibition of PLK1 activity by BI2536 reduced PLK1 intensity at prometaphase kinetochores (Figure 2a). Interestingly, depletion of PICH, by an auxin-inducible degron, together with BI2536 treatment eliminated kinetochore PLK1 (Figure 2a), suggesting that PICH contributes to PLK1 localization to kinetochores when PLK1 self-priming is inhibited. It is possible that the initial PLK1 kinetochore recruitment is dependent on CENP-U, while centromeric PICH serves as an additional receptor to stabilize kinetochore PLK1 (Figure 2b). Since degradation of CENP-U begins in prometaphase [48] while PICH remains localizing to centromeres, we propose that PICH plays an important role to maintain PLK1 at kinetochores in metaphase and anaphase.

(a) RPE1 cells with endogenous *PICH* tagged with mini-auxin inducible degron (mAID) and GFP were first treated with or without doxycycline (Dox) and auxin for 24 h (addition of Dox induces the expression of a E3 ubiquitin ligase TIR1, which destabilizes mAID-tagged protein in the presence of auxin) to induce degradation of PICH-mAID-GFP. Cells were then treated with nocodazole for 2 h and with or without BI2356 (100 nM) for 1 h. GFP, PLK1 and DNA were visualized using anti-GFP antibody (green), anti-PLK1 antibody (red), and DAPI (blue). Images were acquired using a Nikon Ti60 microscope equipped with DS-Ri2 camera. Scale bar, 10 μm.(b) Schematic diagram of the proposed model of PICH serving as an additional receptor to stabilize PLK1 at kinetochores. CENP-U is the key receptor for PLK1. It is phosphorylated by CDK1 and PLK1 to create docking sites for PLK1 binding. CDK1 also phosphorylates PICH to mediate PLK1-PICH interaction. When PLK1 activity is inhibited and PICH is depleted, PLK1 is no longer stably associated with kinetochore. See text for details.

## Different origins of UFBs

**Figure 3.**
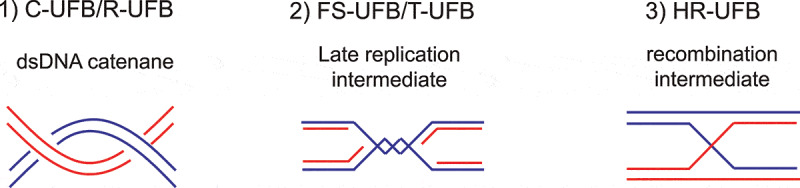
Schematic diagram indicating three major underlying structures of UFBs

UFBs can be classified by either the genomic loci from which they originate or the underlying intertwining structures that they consist of. Despite different origins and structures, it is currently agreed that they are all recognized and processed by the same core UFB-associated proteins, of which PICH is the first and main recruitment factor. Since we focus on how UFBs are resolved by the actions of PICH and its interacting proteins, here we provide the classification of the UFBs by their underlying structures. Three types of UFBs are identified (Figure 3):

Centromeric-UFBs (C-UFBs) originate from centromeres and ribosomal-UFBs (R-UFBs) arise from ribosomal DNA (rDNA) loci. Both C-UFB and R-UFB possess dsDNA catenanes. Fragile site-UFBs (FS-UFBs) emerge from un-replicated DNA at common fragile sites. Telomeric-UFBs (T-UFBs) arise from replication intermediates at telomeres. Finally, homologous recombination-UFBs (HR-UFBs) arise from unresolved recombination intermediates.

*Double-stranded DNA (dsDNA) catenane*. Persistent dsDNA catenanes at centromeres give rise to centromere-UFBs (C-UFBs), which appear in most early anaphase cells [13,14,32,49]. When mitotic cells were treated with a TOP2A inhibitor such as ICRF-193 or ICRF-159, an elevated number of UFBs were observed, supporting the notion that accumulation of dsDNA catenanes lead to UFB formation [13,14,49–51]. In undamaged cells, UFBs are predominantly located at the centromeres, suggesting that DNA decatenation is hindered by the persistence of centromeric cohesion [32]. Apart from C-UFBs, UFBs that arise from catenanes can also be found at ribosomal DNA (rDNA) loci and they are termed ribosomal-UFBs (R-UFBs) [52]. R-UFBs are also commonly found in undamaged anaphase cells. It may be because rDNA requires additional decatenation steps to achieve full condensation and this particular type of DNA is often found with partial catenation even at anaphase [53,54].

*Late replication intermediates*. Common fragile sites (CFSs) are genomic loci that are difficult-to-replicate as they usually contain very large genes that are late replicating and have a low density of replication origins [55]. CFSs are prone to develop cytogenetically defined gaps and breaks on mitotic chromosomes under replication stress conditions, such as upon treatment with a DNA polymerases inhibitor, aphidicolin [55,56]. Un-replicated DNA gives rise to late replication intermediates (LRIs) and subsequently lead to the formation of fragile site-UFBs (FS-UFBs) [57–60]. The termini of FS-UFBs are associated with the FANCD2-FANCI complex, a marker of LRIs, but it is not clear if the complex plays any role in resolving LRIs or it is simply trapped at those regions. Accumulation of un-replicated DNA in mitosis can also be induced by imbalance of the nucleotide pool that impairs DNA replication [61–63]. In BS patient cells, cytidine deaminase (CDA) is downregulated, leading to an excess of deoxycytidine triphosphate (dCTP) and an elevated level of FS-UFBs [61–63]. Furthermore, a recent study showed that treatment of TOP2A inhibitor at the begin of S phase also induced UFBs containing un-replicated DNA as TOP2A inhibition compromises complete DNA replication [64]. Another type of UFBs that also raise from replication intermediates is telomeric-UFBs (T-UFBs). They rarely present in unperturbed cells. Instead, they can be induced by the overexpression of TRF2 (telomeric repeat-binding factor 2) [65], a component of the shelterin complex that protects telomeres from end-to-end fusion [66]. It is proposed that overexpression of TRF2 results in replication stalling at the telomeric regions by exhausting the regulatory system due to the excessive formation of tight DNA-protein complexes. Depletion of BLM induces LRIs accumulation in telomeric regions, therefore also induces T-UFBs formation [67].

*Recombination intermediates*. Double-strand break repair (DSBR) by homologous recombination (HR) often results in the formation of four-way Holliday junctions (HJs) that covalently connect the sister chromatids [68,69]. Two independent groups reported that these HR intermediates can persist at anaphase and give rise to a distinct class of UFBs termed HR-UFBs [70,71]. Importantly, HR-UFBs are not associated with the FANCD2-FANCI complex, and their formation is dependent on the activation of HR pathway. The simultaneous inactivation of two HJ resolvases, GEN1 and MUS81, leads to accumulation of unresolved recombination intermediates and therefore massive HR-UFBs induction [70]. Similarly, depletion of 53BP1, a negative regulator of the HR pathway, also increases the formation of HR-UFBs [71]. In late anaphase, the double-stranded HR-UFBs are converted to RPA-coated, single-stranded bridges that are subsequently broken upon cell division [70]. Elevated level of single-stranded, *FRAXA*-associated UFBs were also observed in fragile X syndrome patient cells with >200 CGG trinucleotide repeats upon folate deficiency [72]. Since accumulation of the *FRAXA*-associated UFBs depends on RAD51, they are believed to represent unresolved recombination intermediates [72].

## UFB recognition and DNA translocation by PICH

Since PICH localizes to UFBs with different underlying structures, it should be able to sense a common characteristic of all UFBs, i.e. DNA stretching by mitotic spindle. This idea is first supported by a study showing that premature loss of centromeric cohesin induced by SGO1 depletion led to a large amount of PICH-decorated UFBs. When cells depleted of SGO1 were incubated with paclitaxel, an inhibitor of MT dynamics, PICH-decorated UFBs were no longer detected [13]. These results indicate that PICH recruitment to UFBs is sensitive to alterations in tension due to the effect of mitotic spindle. Later, a series of single-molecule experiments using microfluidics and optical tweezers determined that PICH displays a higher affinity to dsDNA under tension and cannot bind to single-stranded DNA (ssDNA) [18,73]. This unique property suggests that PICH may act as a primary sensor for recognizing UFBs. Indeed, PICH decorates to all known types of UFBs. Furthermore, most of the other UFB-associated factors (such as RIF1 [74] and the BTRR complex composed of BLM, topoisomerase IIIα, RMI1 and RMI2 [14,19]) depend on PICH for their UFB localization. The single-molecule studies also analyzed the impact of PICH on stretched DNA and concluded that PICH binding is able to generate DNA bending, increase the DNA contour length and stabilize the DNA against unwinding due to overstretching [18]. It is proposed that the stretching of entangled DNA between sister chromatids at anaphase leads to tension-induced nucleosome unwrapping to expose a short region of bare DNA that is then bound and stabilized by PICH. The bound PICH may lower the energy barrier required for further nucleosome unwrapping, which leads to the expulsion of nucleosomes from the entangled DNA, resulting in it being almost entirely coated with PICH [18]. Although PICH was proposed to remodel chromatin by sliding nucleosome, its remodeling activity was found to be very weak [19]. Furthermore, ATPase dead mutant of PICH can still localize to UFBs, suggesting that PICH is unlikely to directly remodel chromatin to remove nucleosomes [18,19,24,51]. On the other hand, replacing wild-type PICH with ATPase dead mutant of PICH leads to persistence of UFBs in telophase, indicating that translocase activity of PICH is important for promoting UFB resolution [51,52].

PICH is able to hydrolyze ATP and utilize the energy gained to displace DNA triplex and promote branch migration of the four-way junction [18]. DNA binding and translocase activity of PICH can be regulated by interaction with other proteins and its SUMOylation. Study of PICH interactome in mitosis determined that other than PLK1 and the known members of UFB-associated proteins, BEN (BANP, E5R, and Nac1) domain-containing protein 3 (BEND3) was also co-precipitated [75]. PICH and BEND3 can interact directly through the interaction of PICH’s N-TPR domain and BEND3’s BD1 domain [75]. BEND3 is a transcriptional repressor that associates with heterochromatin and rDNA loci [76,77]. Importantly, BEND3 enhances the ATPase and translocase activities of PICH [75]. It is possible that BEND3 and PICH translocase activity work together to repress transcription-induced catenation events at rDNA, therefore promoting segregation of sister-rDNA and preventing R-UFBs formation. This possible role of BEND3-PICH has yet to be examined. PICH is also modified by SUMO2/3 on mitotic chromosomes [34]. SUMOylation of PICH is dependent on a mitotic SUMO E3 ligase, protein inhibitor of activated STAT (PIASy), which is a centromeric protein [34]. SUMOylated PICH shows a reduced ability to bind DNA, suggesting that SUMOylation of PICH can regulate its DNA translocase activity. PIASy were also shown to mediate SUMO2/3 modification of TOP2A, which inhibits TOP2A decatenation activity and prevents premature decatenation of centromeric DNA [78].

## Recruitment of other UFB-associated proteins by PICH

PICH serves as the main recruiting factor for the other UFB-binding proteins. The most famous one is BLM [14,19]. BLM interacts with topoisomerase IIIα (TOP3A) and two accessory factors RMI1 and RMI2 to form the so-called BTRR complex, which is well-known for their ability to mediate the dissolution of double HJs [79–84]. Localization of the BTRR complex on UFBs depends absolutely on PICH, but not *vice versa* [14,19]. PICH recruits BLM likely via direct interaction as the C-terminal region of PICH (791–1250 aa.) is necessary for the interaction with BLM [19].

A recent biochemical study has shown that the BTRR complex is able to disjoin two catenated plasmids, which resemble catenated dsDNA in C-UFBs/R-UFBs [73]. Bacterial and yeast orthologs of RecQ/Sgs1-topoisomerase III complexes also show similar decatenation activity [85,86], suggesting that it is an evolutionary conserved activity for this complex. PICH does not increase the dsDNA decatenation activity of the BTRR complex, indicating that PICH’s role is primarily increase the local concentration of the BTRR complex on catenated UFBs [73]. Similarly, the BTRR complex can also disjoin two interlinked gapped plasmids that resemble LRI structure, indicating that PICH also recruits the BTRR complex to FS-UFBs for their subsequent resolution [73]. PICH stimulates the disjoining of the FS-UFB-like DNA substrates in a manner dependent on its ATPase activity, suggesting that PICH plays a dual role at FS-UFBs by both recruiting the BTRR complex and enhancing its enzymatic activity toward LRIs [73]. The BTRR complex is well-known to dissolve double HJs by first catalyzing the convergent migration of two HJs by BLM, forming a hemicatenane that is then unlinked by TOP3A [87]. Therefore, the BTRR complex can process all the proposed underlying structures that give rise to UFBs. Indeed, a marked increase in the frequency of PICH-decorated UFBs is observed in both BLM-deficient and TOP3A-deficient cells [14,19,57,88,89].

Although PICH appears to recruit the whole BTRR complex to UFBs, a recent study showed that BLM and TOP3A-RMI1-RMI2 (TRR) are capable of being recruited independently to PICH-coated UFBs [89]. This was evidenced through the localization of BLM and TRR to UFBs in TOP3A-depleted and BS (Bloom’s syndrome) cells, respectively. However, since the BTRR complex as a whole shows a higher affinity for UFBs than BLM or TRR alone, the BTRR complex is likely to be recruited *in vivo* as a whole to UFBs [89]. However, the possibility of the presence of the PICH-TRR and PICH-BLM subcomplexes that mediate different actions on UFBs cannot be excluded.

Another protein, RIF1, also localizes to UFBs in a PICH-dependent manner [74,90]. RIF1 recruitment to UFBs is independent of BLM, suggesting that RIF1 may play an BLM-independent role at UFBs. Although deletion of PICH-TPR domains (PICH 1–76 aa. and 1090–1250 aa.) fully abrogates its interaction with RIF1 in co-immunoprecipitation assays, its localization to UFBs is not affected, suggesting that the UFB recruitment of RIF1 is not dependent on either direct or indirect interaction with PICH [74]. Furthermore, RIF1 is recruited to UFBs in SGO1-depleted prometaphase cells only upon CDK1 inhibition, indicating that RIF1 recruitment to UFBs is restricted to anaphase in the absence of the cyclin B-CDK1 activity [74]. RIF1 is required for the timely resolution of UFBs as more RPA-decorated UFBs persist during telophase upon depletion of RIF1. Depletion of 53BP1, a protein that recruits RIF1 to DNA damage sites, did not affect RIF1 localization to UFBs [74]. Furthermore, unlike yeast Rif1 that was shown to interact with Rap1 to regulate telomere length [91], there is no clear evidence that human RIF1 is directly involved in telomere function. Therefore, the exact role of human RIF1 in UFBs and potential underlying structures in UFBs that RIF1 binds are still unknown. Since RIF1 is known to interact with protein phosphatase 1 (PP1) and the RIF1-PP1 complex plays multiple roles during the cell cycle [92–97], it is possible that RIF1 recruits PP1 to UFBs to dephosphorylate other UFB-associated proteins, thereby facilitating UFB resolution. This possibility remains to be investigated.

Different UFB-associated proteins are likely to mediate different actions in UFB resolution, and many of them are known to play important roles in other parts of the cell cycle. To differentiate their specific roles on UFBs during anaphase, it is important in the future to employ a robust and inducible system (e.g. auxin-inducible degron system) to inactivate individual UFB proteins at metaphase/anaphase transition. For instance, by using auxin-based degron system to degrade TOP3A, Hickson’s group recently showed that TOP3A facilitates UFB decatenation independent of BLM [73,89].

## Modification of DNA topology and UFB resolution promoted by PICH

**Figure 4.**
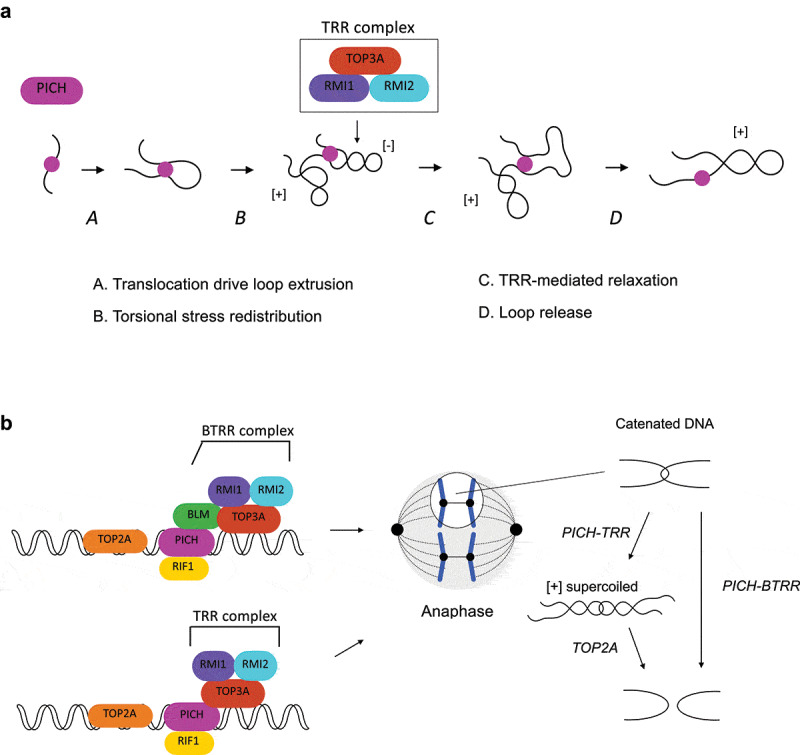
The actions of PICH-BTRR complex toward catenated UFBs

Although PICH alone does not influence DNA topology as it is unable to relax negatively supercoiled DNA [51], a recent biochemical study showed that PICH and the TOP3A-RMI1-RMI2 (TRR) complex are able to act together to induce positive DNA supercoiling in an ATP-dependent manner [89]. A high density of positive supercoiling can be generated by PICH and the TRR complex as indicated by the inability to separate the products by netropsin, a DNA ligand capable of resolving up to ten positive supercoils in agarose gel, and a fully plectonemic conformation observed using atomic force microscopy (AFM) [89]. Positive supercoiling can only be achieved in the presence of PICH translocase activity, as PICH^K128A^ (ATPase-dead mutant) is unable to induce supercoiling when incubated with the TRR complex. How do PICH and the TRR complex corporately induce the positive supercoiling? PICH possesses a DNA loop extrusion activity via DNA translocation that is associated with torsional stress redistribution [89]. In this case, the extruded loop would be hyper-negatively supercoiled while the rest of the DNA is positively supercoiled (Figure 4a). The hyper-negatively supercoiled loop can be subsequently relaxed by the TRR complex, resulting in a positively supercoiled plasmid [89] (Figure 4a). Since positively supercoiled catenanes are TOP2A’s preferred substrates for decatenation [98,99], the PICH-TRR facilitates TOP2A-mediated DNA decatenation. Indeed, depletion of TOP3A increases the frequency of C-UFBs upon ICRF-193 treatment [89]. As mentioned in previous section, the BTRR complex is also capable of disjoining C-UFB-like DNA substrates. Together, these findings indicate that PICH facilitates decatenation of C-UFBs in two independent pathways (Figure 4b). First, PICH acts together with the TRR complex to induce positive supercoiling to facilitate TOP2A-mediated decatenation. Second, PICH recruits the BTRR complex and increases the local concentration of this complex on C-UFBs, where the BTRR complex mediates decatenation. The reason for the need of two pathways is unknown. The BTRR complex can simply serve as the backup system to rescue anaphase when TOP2A is compromised. However, depletion of BLM/TOP3A significantly increases the amount of UFBs in TOP2A proficient cells [14,19,57,88,89], suggesting that the BTRR complex-mediated decatenation pathway is indispensable. One possible explanation is that since the BTRR-mediated decatenation pathway does not depend on the positive supercoiling of DNA, TOP2A and the BTRR complex target different pools of C-UFBs dependent on whether positive supercoiling can be induced.

(a) Schematic diagram showing the proposed model for how positive supercoiling is introduced by PICH and the TRR complex. PICH catalyzes the loop extrusion associated with torsional stress redistribution. The extruded loop is negatively supercoiled while the rest of the DNA is positively supercoiled. The negatively supercoiled loop can be subsequently relaxed by TRR, resulting in a positively supercoiled plasmid. See text for details.(b) PICH facilitates C-UFB resolution by recruiting the BTRR complex that mediates decatenation to UFBs. PICH also acts together with TRR to induce positive supercoiling that facilitates TOP2A-mediated decatenation.

## PICH and cancer

Over the years, numerous studies showed that the loss of UFB-associated proteins results in chromosome segregation defects and subsequent genome instability that manifest as the formation of chromatin bridges, 53BP1 foci and micronuclei [14,19,23,24,51,74,88,100]. Similarly, *Pich* knockout induces early embryonic lethality in mice due to a global increase in DNA damage and p53 activation [100]. Importantly, RAS activation together with overexpressing E1A induce transformation of wild-type MEFs but *Pich* heterozygous MEFs is refractory to RAS/E1A-induced transformation, suggesting that PICH is important to support the rapid proliferation of transformed cells [100]. Consistent with this notion, PICH expression was found to be significantly higher in cancer patient samples with *p53* truncating mutations [100]. It is therefore not surprising that several recent studies have implicated the role of *PICH* in tumorigenesis. For instance, PICH was found to be significantly overexpressed in various cancers, particularly in breast and kidney cancers [101,102]. Likewise, upregulation of *PICH* has also been found to associate with the progression of tumor and poor prognosis in hepatocellular carcinoma [103–105]. These findings are consistent with the idea that PICH is needed to support the proliferation of rapidly growing cancer cells. Indeed, silencing of *PICH* in triple negative breast cancer, kidney cancer and hepatocellular carcinoma results in the suppression of tumor growth in xenograft mice model [101,102,105]. Together, these results suggest that the PICH can act as a prognosis indicator and a promising therapeutic target in many different cancers.

## Concluding remarks

In prometaphase, PICH localizes to chromosome arms and centromeres where it remodels SUMOylated chromosomal proteins for proper organization of chromosomes. Recent studies have shown that PLK1 negatively regulates PICH/BLM at centromeres. On the other hand, PICH may serve as an additional receptor for PLK1 at kinetochores. In anaphase, PICH senses DNA tension from separating sister chromatids that are still connected by topological linkages, which manifest as UFBs. Binding of PICH to UFBs further recruits other UFB-binding proteins, such as the BTRR complex and RIF1 to the bridges, and together they mediate UFB resolution. Future research should focus on how phosphorylation mediated by PLK1 on the PICH-BTRR complex affects both its localization and activity on chromosomes and UFBs. Furthermore, PICH is implicated in maintaining genome stability. Since rapidly growing cancer cells usually exhibit replication stress and high level of endogenous DNA damage, they are expected to accumulate replication/recombination intermediates that lead to elevated level of UFBs. We speculate that cancer cells with increased frequency of UFBs rely on PICH-mediated UFB resolution pathway to sustain proliferation. Therefore, the potential of PICH as a therapeutic target in cancers should be explored in the future.
